# Xylo-Oligosaccharides, Preparation and Application to Human and Animal Health: A Review

**DOI:** 10.3389/fnut.2021.731930

**Published:** 2021-09-08

**Authors:** Yuxia Chen, Yining Xie, Kolapo M. Ajuwon, Ruqing Zhong, Tao Li, Liang Chen, Hongfu Zhang, Yves Beckers, Nadia Everaert

**Affiliations:** ^1^State Key Laboratory of Animal Nutrition, Institute of Animal Science, Chinese Academy of Agricultural Sciences, Beijing, China; ^2^Precision Livestock and Nutrition Unit, Gembloux Agro-Bio Tech, TERRA Teaching and Research Centre, Liège University, Gembloux, Belgium; ^3^School of Life Science and Engineering, Southwest University of Science and Technology, Mianyang, China; ^4^Departments of Animal Sciences, Purdue University, West Lafayette, IN, United States; ^5^Hunan United Bio-Technology Co., Changsha, China

**Keywords:** xylo-oligosaccharides, preparation, application, human health, animal health

## Abstract

Xylo-oligosaccharides (XOS) are considered as functional oligosaccharides and have great prebiotic potential. XOS are the degraded products of xylan prepared via chemical, physical or enzymatic degradation. They are mainly composed of xylose units linked by β-1, 4 bonds. XOS not only exhibit some specific physicochemical properties such as excellent water solubility and high temperature resistance, but also have a variety of functional biological activities including anti-inflammation, antioxidative, antitumor, antimicrobial properties and so on. Numerous studies have revealed in the recent decades that XOS can be applied to many food and feed products and exert their nutritional benefits. XOS have also been demonstrated to reduce the occurrence of human health-related diseases, improve the growth and resistance to diseases of animals. These effects open a new perspective on XOS potential applications for human consumption and animal production. Herein, this review aims to provide a general overview of preparation methods for XOS, and will also discuss the current application of XOS to human and animal health field.

## Introduction

**Figure 1:**
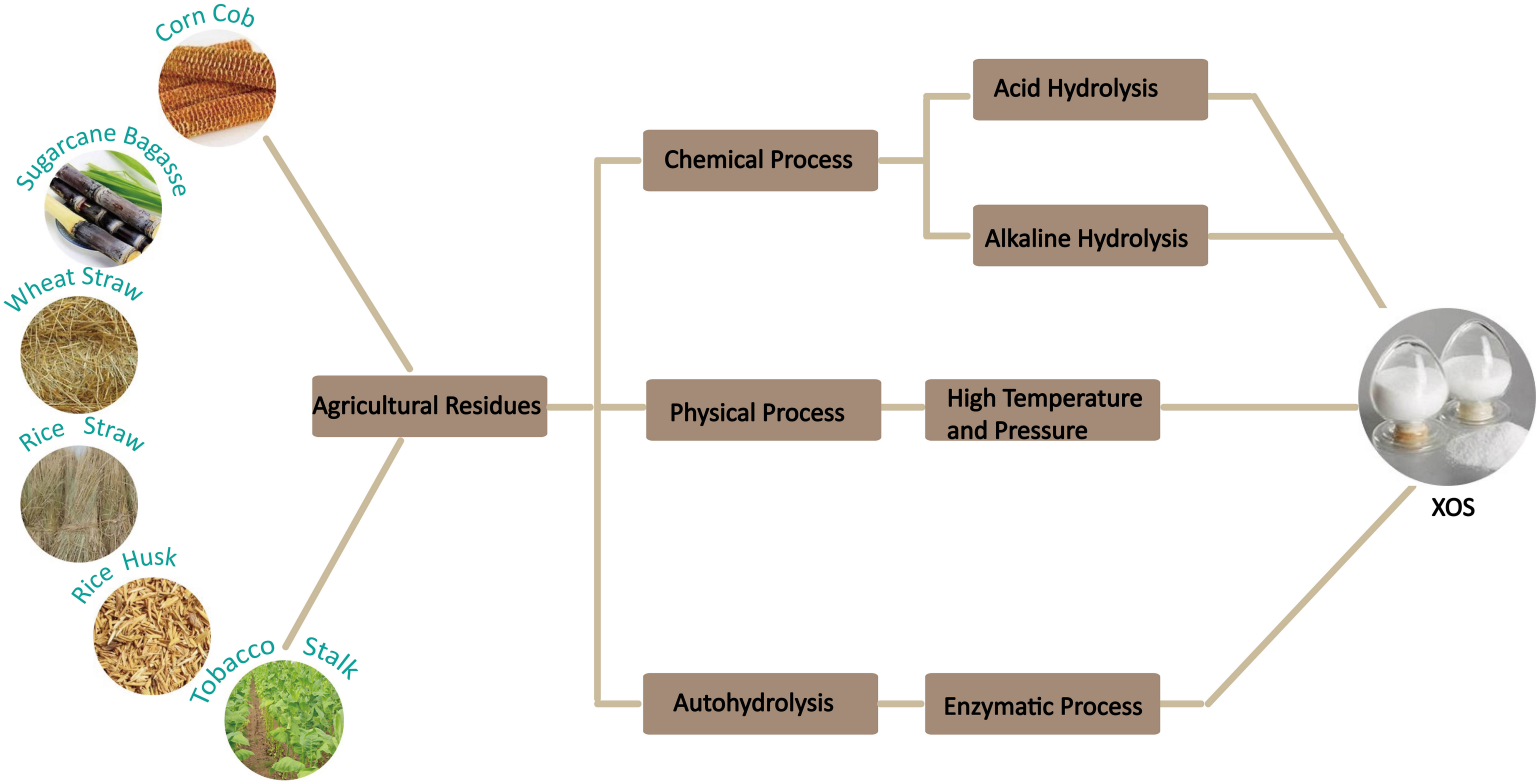
Schematic of XOS production from agricultural residues.

**Figure 2:**
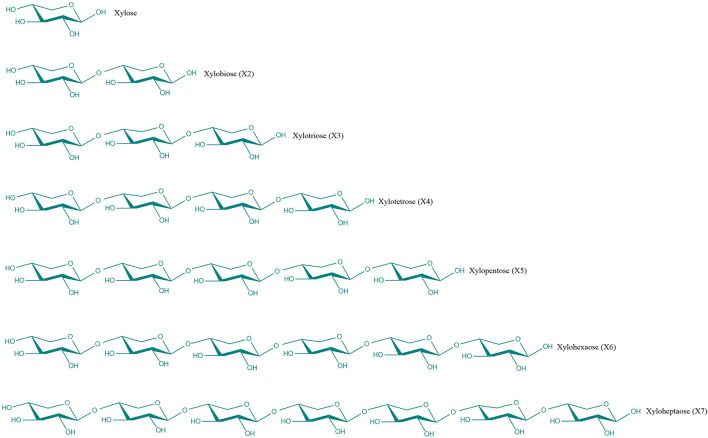
Chemical schematic structure of XOS with low degree of polymerization.

During the few last decades, there is increasing interest in the use of nutraceuticals or functional food additives for improving human health which has led to development of new food and feed products during the last few decades (1). Many functional products, having prebiotic characteristics, such as xylo-oligosaccharides (XOS), fructo-oligosaccharides (FOS), galacto-oligosaccharides (GOS), chitooligosaccharides (COS), alginate-oligosaccharides (AOS) have been extensively used as food and feed additives (2–6). Among these prebiotics, XOS are considered to be very promising. XOS are the degraded products prepared by chemical, physical or enzymatic degradation of xylan derived from biomass materials such as sugarcane residues, corn cobs, rice straw, etc (7) (Figure 1). They are composed of xylose units linked by β-1, 4-xylosidic bonds, which have a branched structure by the addition of different side groups (Moreira et al.). The degrees of polymerization of XOS are usually 2–7 (Figure 2) and they are known as xylobiose, xylotriose, and so on (8).

XOS have a high potential to be applied for human nutrition due to its physicochemical properties such as low viscosity, high water solubility, tolerance to high temperature and acidic pH (9). Studies shown that XOS display a variety of pharmacological activities, including anti-inflammation, antioxidative, antitumor, antimicrobial properties. In addition, XOS have a potential application in the animal husbandry (10, 11). This review aims to summarize the methods of preparation of XOS and discuss the application of XOS to human and animal health.

## Preparation and Characterization of XOS

**Figure 3:**
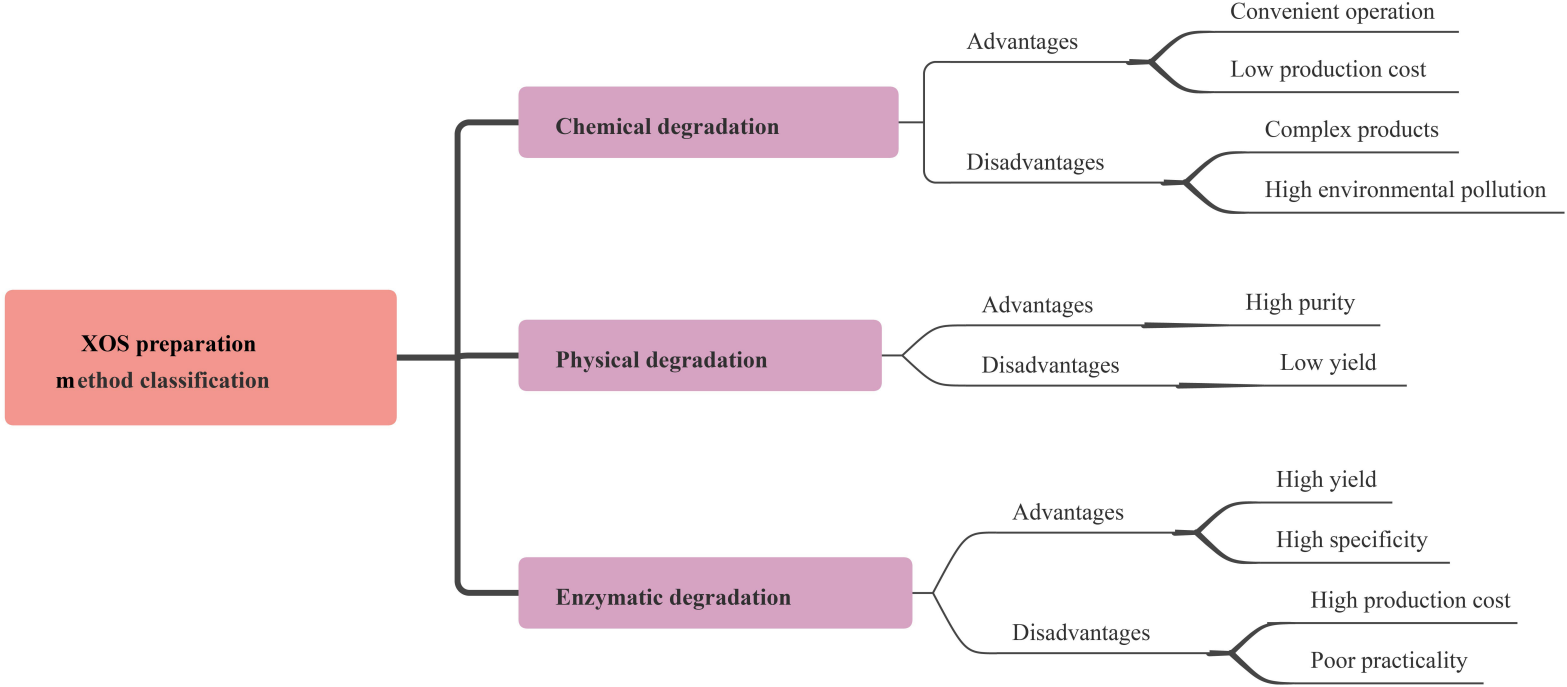
Characterization of XOS preparation methods.

The most widely used preparation methods of XOS are: (1) chemical degradation methods (2) physical degradation methods and (3) enzymatic degradation methods (Figure 3).

### Chemical Process for the Production of XOS

The chemical degradation process, especially the acid and the alkaline hydrolysis methods, has been widely used for the mass production of XOS in industry due to its advantages such as simple operation and low production cost. Several studies have been conducted on producing XOS with various inorganic acids (12–16). Samanta et al. reported that the xylan from tobacco stalks was hydrolysed by tartaric acid into XOS, mainly including xylobiose and xylotriose, in addition to monomeric xylose (16). XOS production can also be obtained from corn cob xylan using weak sulphuric acid at 90°C during 30 min (12). The production of XOS depends on both acid concentration and hydrolysis time. A previous study showed that optimization of XOS production from waste xylan optimized by an orthogonal design of experiments, concluding a good extraction procedure of 20 min with 20% acetic acid at 140°C. A maximum XOS yield of more than 45.86% was obtained (14). Ying et al. studied that the increment of sulfuric acid concentration promoted the yield of xylooligosaccharides from hydrogen peroxide-acetic acid-pretreated poplar from 0.69 to 20.45% (17). In addition, Zhang et al. reported that acetic acid hydrolysis provided the highest XOS yield, up to 45.91% compared to hydrochloric acid and sulfuric acid pretreatment (15). It is widely known that the alkali solution could degrade hemicelluloses. This destruction is caused by the disruption of the hydrogen bonds with the alkaline reagent (18). In order to enhance the xylan content recovery from hemicellulose, use of appropriate alkaline concentration and pretreatment parameters are the primary conditions (19). For example, the use of higher concentration of alkali solution (15%) for extracting pineapple peels led to maximum recovery of hemicellulose. In the case of corn cobs, Samanta et al. also documented that higher concentration of alkali produced greater dissolution of hemicelluloses (12). However, these methods caused corrosion of the equipment, thus limiting their use.

### Physical Process for the Production of XOS

Production XOS products by physical degradation is relatively simple and environmentally friendly compared to chemical degradation. For example, XOS can be obtained from milled aspen wood using a microwave oven, processing at 180°C for 10 min were and nextly subjected to fractionation to oligo- and polysaccharides by size-exclusion chromatography. The dispersion degree was smaller while the degradation effect was better (20). The hydrothermal reactor can also be used to degrade the xylan. Its fragments released from corn cob hemicellulose are partially acetylated, which improves solubility of long xylo-oligosaccharides by preventing molecular interactions between the xylan and the main chains of the xylo-oligosaccharide and also by preventing the binding of xylan to cellulose (21). The purity of XOS products is relatively high from physical degradation. However, there is limitation on the use of this method for large-scale production of XOS due to low yield.

### Enzymatic Process for the Production of XOS

The industrial process of XOS production from natural xylan-rich agricultural residues involve enzymatic hydrolysis. As compared to the acid and alkaline hydrolysis method, production by the enzymatic degradation is relatively more economical, quick, and eco-friendly. Furthermore, enzymatic hydrolysis neither requires any special equipment nor produces undesirable byproducts. Thus, the production of XOS by enzymatic means was done from plant sources rich in xylan including corn cobs, sugarcane bagasse, wheat bran, birch wood, oat spelt, beech wood, natural grass, oil palm frond etc. These major enzymes used include β-xylosidase, glycosynthases and endo-xylanases, the latter being the key enzyme to produce XOS from xylan. They are able to reduce monomeric xylose release from the non-reducing ends of xylooligomers and xylobiose. The endo-xylanases from families GH10, GH11, and GH30 act specifically on the substituted and unsubstituted regions of xylan chain (22). Other studies focused on the use of β-xylosidases and glycosynthases for XOS production. β-xylosidases catalyze subtrate hydrolysis by inversion or retaining mechanism and are classified into six GH families: GH3, 30, 39, 43, 52, and 54. The β-xylosidases have been reported to produce longer β-XOS from β-1, 4 linkages or synthesize novel XOS (23, 24). Kim et al. documented that a glycosynthase derived from a retaining xylanase could synthesize a great variety of XOS (25). Many factors affect the yield of XOS from xylan such as the enzyme activity, the raw material, and incubation conditions including incubation pH, reaction time and temperature (19).

Table 1 summarizes the preparation process and the yields of XOS produced from xylan and xylan biomass by different approaches, often leading to high yields for several sources of substrates. Importantly, the prebiotic action of XOS requires a low degree of polymerization (DP) (9, 18). Hence, there are still some parameters in the preparation process of XOS that need to be optimized, including the production of a low DP (DP of 2–7) and the achievement of a high purity. Therefore, research focuses on the combination and integration of the processes, testing different raw materials, extraction methods and enzymes to achieve an economically viable and health promoting product with an optimal production efficiency.

**Table 1:** Summary of XOS preparation and yields in the most recent studies.

**Substrate**	**Pretreatment**	**Biocatalyst**	**Yxylan/biomass (%)**	**Yxos/biomass (%)**	**Yxos/xylan (%)**	**DP**	**References**
Corn cobs	acetic acid pH 2.7, 150°C, 30 min		30.4%	13.97%	45.9%	X2-X6	(14)
	Dilute acid followed by 135°C for 30 min	Xylanase from Penicillium corylophilum P-3-31	34.8%	23.6%	67.7%	X2-X4	(26)
	pH 6.5 and 60°C	Xylanase (PbXyn10A)	31.2%	23.4%	75%	X2-X4	(27)
	ultra-high pressure pretreatment	Streptomyces thermovulgaris TISTR1948 endoxylanase	33.4%	3.6%	10.7%	X2-X4	(28)
	190 C, 13 min	GH10 xylanase	29.9%	14.8%	49.4%		(21)
	5% (w/v) KOH, 90°C for 1 h		38.8%	11.5%	29.6%	X2-X5	(29)
Sugarcane Bagasse	Alkaline 10% (w/v) at room temperature overnight	Endo-β-1,4-xylanase rHlxyn11A	10.5%	6.0%	57.4%	X2-X3	(30)
	15% (w/v) aqueous ammonia	β-xylosidase	28.40%	19.3%	68.0%	X2-X4	(31)
	0.24M dilute H_2_SO_4_ 90 C 31 min		33.5%	9.7%	29%	X2-X6	(13)
	5% gluconic acid hydrolysis (w/v) 60 min at 150°C	Cellulase	26.5%	14.1%	53.2%	X2-X6	(32)
	10% acetic acid at 150°C for 45 min	G. oxydans ATCC 621H	27.9%	10.9%	39.1%	X2-X6	(33)
Wheat straw	2% NaOH at 80°C for 90 min	The endoxylanase-variant K80R	8.4%	3.3%	39.8%	X2-X3	(34)
	Hydrolysis at 50°C and pH 5 for 5 h	β-1,4-endoxylanase			44%	X2-X3	(35)
	180°C 40 min	Endo-β-1-4-xylanase	73%	23%	31.5%	X2-X3	(36)
Rice straw	2% w/w sulfuric acid, 100°C, 0.5 h		65.3%	18.2%	27.8%		(37)
Rice husk	12% w/v NaOH, 110–120°C for 30 min	β-1,4-xylanase	54.5%	9.5%	17.4%	X2-X5	(38)
Pineapple peel	15% (w/v) alkali solution for 16 h at 45°C, 50°C, pH 5.0 and 15 U enzyme dose	Endo- 1, 4–Xylanase M1		23.5%	25.7%	X2-X3	(39)
Finger millet seed coat	Sodium acetate	Xylanase of Thermomyces lanuginosus	4.8%	3.4%	71.8%	X2-X3	(40)
Tobacco stalk	8% KOH or NaOH 90°C, 1M tartaric acid		17.0%	6.1%	35.7%	X1-X3	(16)

## XOS Application to Human Health

**Figure 4:**
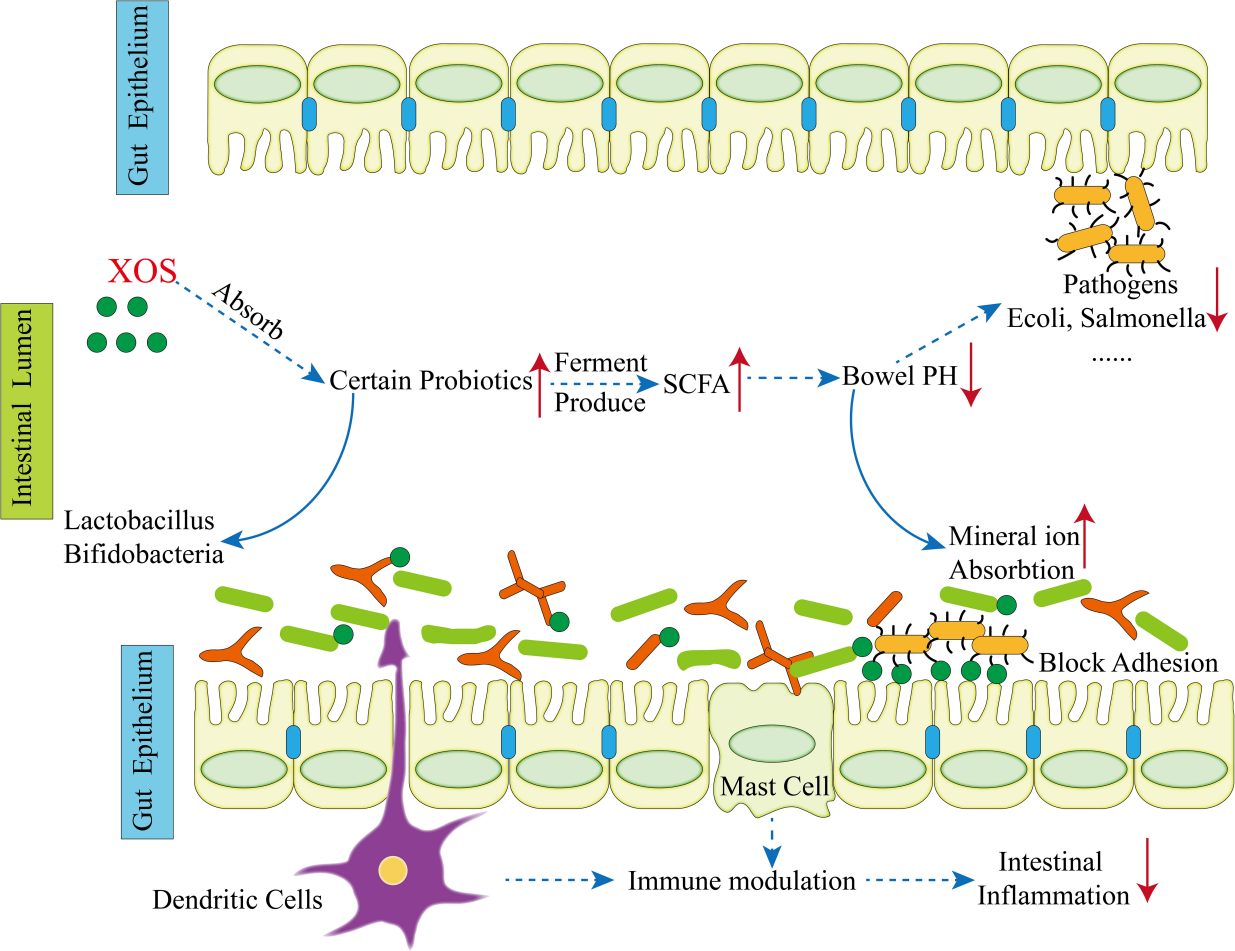
Potential health benefits of prebiotics [quoted from (41)].

XOS were demonstrated to have various activities in human health such as inducing immune modulation, anti-tumor, antioxidant and anti-microbial effects (Figure 4).

### Immune Modulation Effects of XOS

It is essential for protecting the host from diseases or repairing tissue injury to release inflammatory mediators (42, 43), and XOS is thus suggested to be an immunomodulator to prevent adverse immune-related conditions. Indeed, XOS was shown to have immunomodulatory effects by regulating expression of several proinflammatory mediators *in vitro*. XOS not only suppressed TNF-α, IL-1β, IL-6 and NO expression, but also triggered IL-10 production in lipopolysaccharide (LPS)-stimulated RAW264.7 cells (44). XOS feeding significantly decreased expression of IL-1β and IFN-γ and attenuated systemic inflammation (45). Moreover, the O-acetylated XOS derived from almond shells and their deacetylated derivatives exhibited immunomodulatory potential, based on a mitogenic rat thymocyte test (46). Finally, XOS combined with inulin attenuated the expression of IL-1β in the blood of healthy subjects fed a high-fat diet (47). Schematic presentation of XOS health benefits and their role in immune modulation are depicted in Figure 4.

### Anti-tumor Effects of XOS

The main causes of cancer are the uncontrolled proliferation of abnormal cells which may stay at the point of mutation or metastasize into other locations. It has been shown that XOS exposure showed effect in preventing cancer (48–50). Indeed, β-1,3-Xylooligosaccharides with an average DP of 5 extracted from green alga Caulerpa lentillifera inhibited the number of viable human breast cancer MCF-7 cells in a dose-dependent manner, and induced apoptosis (50). Thus, this XOS could be a promising agent for prevention of breast cancer. Moreover, XOS supplementation reduced the level of lipid peroxidation and increased the activities of glutathione-S-transferase and catalase in colonic mucosa and liver, which may have contributed to the inhibition of colon carcinogenesis (51). *In vitro* approaches will be useful for future mechanistic characterization of the antitumor properties of XOS. However, no systematic attempts have been carried out to study the upstream signals of caspase activation and the specific effects *in vivo*. Further research is necessary to investigate the overall anti-tumor effect of XOS.

### Antioxidant Effects of XOS

During both acute and chronic diseases in humans, the abundance of free radicals usually increases. Several notable studies demonstrated that XOS had exhibited strong antioxidant and free radical scavenging activity, thus suggesting a potential use in biomedical applications (52, 53). The scavenging ability of XOS was shown to be dose-dependent (54), and this potential is likely attributable to efficient release of phenolic compounds and transfer of hydrogen atoms from the phenolic compounds to free radicals (55). Jagtap et al. revealed that the percent of antioxidant activity gradually increased reaching the maximum, 74% at a concentration of 6 mg/ml XOS using 1,1-diphenyl-2-picryl-hydrazyl (DPPH) assay, after which it did not show any further increase (56). Bouiche et al. studied that the antioxidant activity of glucuronoxylooligosaccharides (UXOS) and arabinoxylooligosaccharides (AXOS) was tested with the 2, 2′-Azino-bis (3-ethylbenzothiazoline-6-sulfonic acid) (ABTS) method (57). The results showed that the antioxidant activity of UXOS was significantly higher than the antioxidant activity of AXOS. Although both have neutral molecules, UXOS also has methylglucuronic acid (MeGlcA) decorations that confer a negative charge to the XOS. It was assumed that the MeGlcA decorations of the XOS were key elements influencing their antioxidant and radical scavenging activity of XOS (58).

### Anti-microbial Effects of XOS

It has been reported that XOS have significant antimicrobial effects against several pathogenic bacterial. A host of clinically important both Gram-negative and Gram-positive bacteria have been documented to be sensitive to XOS exposure. Indeed, XOS and FOS supplementations markedly reduced the cecal pH level and increased the population of bifidobacterial compared with the control and DMH (1,2-dimethylhydrazine) treatments and the XOS treatment group had a lower abundance of *E. coli* than the DMH group. These results indicated that XOS and FOS non-digestible carbohydrates may promote the health of intestinal tract (59). In addition, some *in vitro* studies have documented that XOS supplementation produced lactic acid and acetic acid, which contributed to growth of *bifidobacteria* and *lactobacilli* strains and inhibited the growth of pathogenic strains (60–63).

## XOS Application to Animal Health

**Figure 5:**
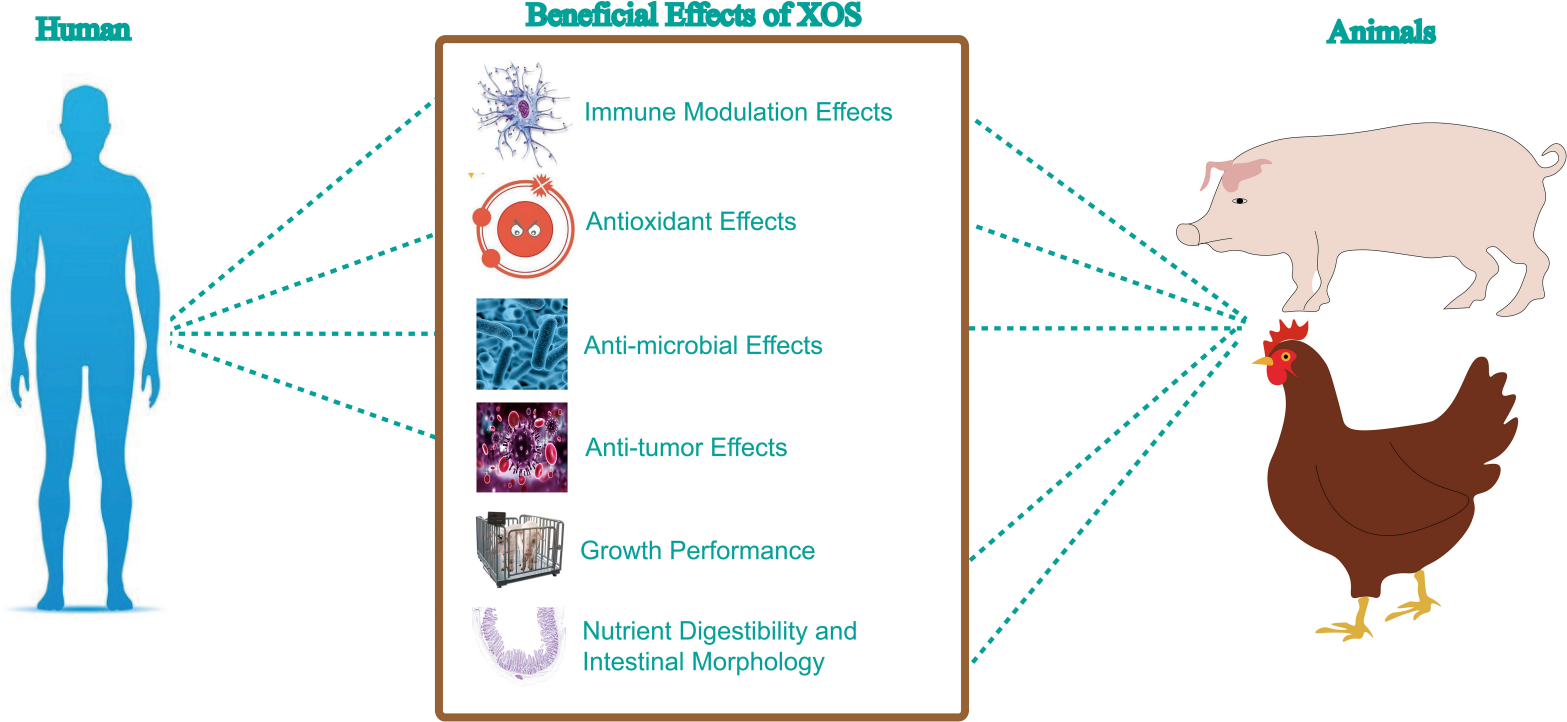
Health beneficial effects of XOS.

In this section, the recent studies on the application of XOS in animal husbandry health are provided. We have noted that most of the studies were focusing on XOS modulation of growth performance, nutrient digestibility and intestinal morphology, immune and anti-oxidant activity and gut microbiome (Figure 5).

### Effects of XOS on Growth Performance of Animals

XOS have been used for animal nutrition and health improvement due to their potential biological functions, such as antioxidant, anti-inflammatory and antimicrobial effects. Previous studies have demonstrated the benefits of XOS on the growth performance of animals. Liu et al. reported that XOS treatment at a dose of 200 mg/kg increased average daily gain (ADG) by 17% and gain to feed (G/F) by 14% in the whole experiment, improved the apparent total tract digestibility (ATTD) of dry matter (DM), N and gross energy (GE) during 0 to 14 d in the piglets (27). Our study found that the effects of 500 mg/kg XOS (XOS500) on the growth performance during 1–28 days were very similar with that of the antibiotic chlortetracycline in the piglets. The results showed that XOS500 (500 mg/kg XOS) supplementation could significantly increase body weight (BW), ADG, average daily feed intake (ADFI) and feed to gain (F: G) of piglets (64). However, another study failed to notice significant improvement on growth performance after 0.01% XOS treatment in pigs (65). The discrepancy might be caused by the different levels of XOS used in these studies. Thus, further studies are needed to confirm the optimal dose of XOS in pigs. In addition, Yuan et.al evaluated the effects of XOS on growth performance and immune function of broiler chickens. They reported that XOS supplementation in the diet of broiler chickens significantly improved ADFI and ADG at 1–42 days when compared to the control group (66). The results of a study by Pourabedin et al. demonstrated that the feed conversion ratio (FCR) in broilers fed 2 g XOS/kg diet was lower than those fed 1 g XOS/kg diet between days 7 and 21, which is in line with other studies (67, 68). Some other researchers found that the FCR in the control group was also significantly lower for the group receiving the XOS-supplemented diet in broiler chickens for the whole trial period (67, 68). These results showed that XOS may dose-dependently improve the growth performance of animals and have potential as novel alternatives to antibiotics as growth promoters.

### Effects of XOS on Nutrient Digestibility and Intestinal Morphology of Animals

The growth promoting effect of XOS has been shown to be related to improvement in nutrient digestibility. The addition of 200 mg/kg XOS with a purity of 50% supplementation has been demonstrated to improve the apparent total tract digestibility (ATTD) of dry matter (DM), nitrogen (N), and gross energy (GE) in weaning pigs on d14 (11). Similarly, the XOS supplementation significantly increased the apparent digestibility of the calcium with the increasing concentration of dietary XOS (0.1, 0.2, 0.3, 0.4 or 0.5 g/kg) in laying hens (69). The improvement of nutrient digestibility may be the result from XOS supporting normal intestinal morphology. Intestine morphology indices are often as a useful criterion to estimate the nutrient digestion and absorption capacity of the intestine. It is generally believed that the jejunum is the main segment involved in absorption of nutrients and minerals (70). Our study indicated that the XOS500 supplementation increased the villus height and villus height to crypt depth ratio in the jejunum and ileum in comparison with the CON and XOS1000 group in the piglets, possibly improving nutrient absorption (71). Liu et al. confirmed that the XOS increased villus height to crypt depth ratio in jejunum, but did not influence villus height, crypt depth in the piglets (27). Similarly, Ding et al. reported that there was a linear improvement in villus height and villus height to crypt depth ratio of the jejunum as dietary XOS concentration increased in the laying hens (10). This is in agreement with the study of Maesschalck et al. showing that supplementation of 0.5% XOS with a purity of 35% to broiler chicken feed significantly increased the villus height in the ileum, suggesting an increase in gut health and improved nutrient absorption (68). However, 0.01% XOS with a purity of 40% in the diet of weaned piglets had little effects on the intestinal structure and villus surface area (65). In addition, the addition of 75 mg/kg XOS with a purity of 35% in the diet decreased the crypt depth of the duodenum (67). These results indicated that the use of an appropriate level of XOS may be important for increasing intestinal health and function.

### Effects of XOS on Immune Modulation and Anti-oxidant Activity of Animals

XOS have been reported to display significant anti-inflammatory and anti-oxidant activities in animals in previous studies. In pigs, Yin et al. reported that dietary XOS markedly reduced serum IFN-γ concentration, indicating an anti-inflammatory effect of XOS (65), which is in line with a study in broilers showing a downregulation of the IFN-γ gene mRNA expression of jejunal mucosa. In addition, an increase in plasma IgG concentration was observed in XOS-fed 21-day-old broilers (66). Furthermore, XOS increased plasma IgA, IL-2, and TNF-α concentration compared with the control diet, and linearly improved the IgA and TNF-α concentration in plasma increasing the dietary XOS concentration in the laying hens (10). These results indicated that dietary XOS may improve cell-mediated immune response in early weaned piglets by regulating the production of cytokines and antibodies. In addition, antioxidant defense systems are regarded as important serum indices for assessing animal health. The changes in the antioxidant defense systems mainly including total antioxidant capacity (T-AOC), total superoxide dismutase (T-SOD), catalase (CAT) and glutathione peroxidase (GSH-Px) may indicate oxidative stress (72). Several studies revealed that XOS had exhibited antioxidant and radical scavenging competency (73). However, the research of Guerreiro revealed that the XOS supplementation reduced antioxidant enzyme activities in European sea bass (74).

### Effects of XOS on the Modulation of Gut Microbiome of Animals

Our recent study showed that XOS500 supplementation could significantly increase the relative abundance of *Lactobacillus* genus and reduce the relative abundance of *Clostridium_sensu_stricto_1, Escherichia-Shigella*, and *Terrisporobacter* genus in the ileum and cecum in piglets (64). Moreover, 200 mg/kg XOS administration decreased fecal *Escherichia coli* and increased *Lactobacilli* in piglets (11). However, dietary XOS reduced the relative abundance of the *Lactobacillus* and increased the relative abundances of *Streptococcus* and *Turicibacter* (65). Furthermore, XOS and GOS both markedly decreased the numbers of intestinal *Listeria* monocytogenes in ileal samples from guinea pigs, and selectively stimulated *bifidobacteria* and *lactobacilli*, which are believed to have inhibitory effects against pathogens (75). Similar beneficial effects of XOS have been observed in broilers. Indeed, 2 g XOS/kg diet increased the relative abundance of the *Lactobacillus* genus in the cecal microbiota of broilers (76), that can adhere to the mucosa and epithelium, promoting colonization, immunomodulation and protecting the intestinal barrier against pathogens (77). Furthermore, by the production of lactate, the lower the intestinal pH, inhibiting the growth of acid-sensitive pathogenic bacteria (78). However, the specific effect mechanism of XOS on the gut microbiome remains unclear as several studies were only done (18–20) or by microbial culture methods (21) that fail to provide accurate taxonomic composition and community structure. Thus, extensive research will be required to determine effects of XOS on the microbiome in animals.

## Conclusion

In this review paper, we have summarized the preparation methods for XOS and its potential use as a functional food or feed additive for human and animal health. XOS seem to beneficially promoting intestinal health by selective stimulation of growth of *bifidobacteria* and *lactobacilli*. XOS also reduce the abundance of potentially pathogenic organisms. In addition, XOS exhibit a variety of biological activities including effects in suppressing inflammation, antioxidative, antitumor and antimicrobial properties. However, there are still several bottlenecks in the preparation and application of XOS. It is still difficult to obtain XOS products in large scale with high purity, and lack of consistency in quality of different batches of XOS from different polymerization degrees due to a lack of standardized preparation methods. The XOS products in the market are mainly mixtures not monomers. Technologies should be developed for producing XOS monomers with high purity at low cost. In addition, new investigations are required to further elucidate the specific molecular mechanisms of XOS. Additional information is needed on the mode of absorption of XOS in the host after oral ingestion, and the identification of related receptors or responsible for the transportation of XOS into target cells. Progress in these areas may enhance the value of XOS for applications in the prevention and treatment of human diseases and animal production.

## Author Contributions

YC, LC, and HZ wrote the first draft of the manuscript. NE, YB, and KA critically evaluated the manuscript. YX, RZ, and TL help check and revise the manuscript. All authors contributed to the article and approved the submitted version.

## Conflict of Interest

TL is employed by Hunan United Bio-technology Co. The remaining authors declare that the research was conducted in the absence of any commercial or financial relationships that could be construed as a potential conflict of interest.

## Publisher's Note

All claims expressed in this article are solely those of the authors and do not necessarily represent those of their affiliated organizations, or those of the publisher, the editors and the reviewers. Any product that may be evaluated in this article, or claim that may be made by its manufacturer, is not guaranteed or endorsed by the publisher.

## References

[1] Bitzios M Fraser I Haddock-Fraser J. Functional ingredients and food choice: results from a dual-mode study employing means-end-chain analysis and a choice experiment. Food Policy. (2011) 36:715–25. 10.1016/j.foodpol.2011.06.004

[2] Samanta AK Jayapal N Jayaram C Roy S Kolte AP Senani S. Xylooligosaccharides as prebiotics from agricultural by-products: production and applications. Bioact Carbohydr Diet Fibre. (2015) 5:62–71. 10.1016/j.bcdf.2014.12.003

[3] Bali V Panesar PS Bera MB Panesar R. Fructo-oligosaccharides. Production, purification and potential applications. Crit Rev Food Sci Nutr. (2015) 55:1475–90. 10.1080/10408398.2012.694084 24915337

[4] Canfora EE Van D Hermes G Goossens GH Jocken J Holst JJ. Supplementation of diet with galacto-oligosaccharides increases bifidobacteria, but not insulin sensitivity, inobeseprediabetic individuals. Gastroenterology. (2017) 153:87–97. 10.1053/j.gastro.2017.03.051 28396144

[5] Yuan X Zheng J Jiao S Cheng G xFeng C Du Y. A review on the preparation of chitosan oligosaccharides and application to human health, animal husbandry and agricultural production. Carbohydr Polym Sep. (2019) 220:60–70. 10.1016/j.carbpol.2019.05.050 31196551

[6] Liu J Yang S Li X Yan Q Jiang Z. Alginate oligosaccharides: production, biological activities, potential applications. Compr Rev Food Sci Food Saf. (2019) 18:1859–81. 10.1111/1541-4337.12494 33336967

[7] Jain I Kumar V Satyanarayana T. Xylooligosaccharides: an economical prebiotic from agroresidues and their health benefits. Indian J Exp Biol. (2015) 53:131–42. Available online at: http://nopr.niscair.res.in/handle/123456789/30744 25872243

[8] Aachary AA Prapulla SG. Xylooligosaccharides (XOS) as an emerging prebiotic: microbial synthesis, utilization, structural characterization, bioactive properties, and applications. Compr Rev Food Sci F. (2011) 10:2–16. 10.1111/j.1541-4337.2010.00135.x

[9] Wei L Yan T Wu Y Chen H Zhang B. Optimization of alkaline extraction of hemicellulose from sweet sorghum bagasse and its direct application for the production of acidic xylooligosaccharides by bacillus subtilis strain MR44. PLoS ONE. (2018) 13:e0195616. 10.1371/journal.pone.0195616 29634785 PMC5892927

[10] Ding XM Li DD Bai SP Wang JP Zeng QF Su ZW. Effect of dietary xylooligosaccharides on intestinal characteristics, gut microbiota, cecal short-chain fatty acids, and plasma immune parameters of laying hens. Poult Sci. (2018) 97:874–81. 10.3382/ps/pex372 29294100

[11] Liu J Cao S Liu J Xie Y Zhang H. Effect of probiotics and xylo-oligosaccharide supplementation on nutrient digestibility, intestinal health and noxious gas emission in weanling pigs. Asian Austr J Anim Sci. (*2*018a) 31:1660–9. 10.5713/ajas.17.0908 29642680 PMC6127592

[12] Samanta A Senani S Kolte AP Sridhar M Sampath K Jayapal N. Production and *in vitro* evaluation of xylooligosaccharides generated from corn cobs. Food Bioprod Process. (2012) 90:466–74. 10.1016/j.fbp.2011.11.001

[13] Bian J Peng P Peng F Xiao X Xu F Sun. -C. Microwave-assisted acid hydrolysis to produce xylooligosaccharides from sugarcane bagasse hemicelluloses. Food Chem. (2014) 156:7–13. 10.1016/j.foodchem.2014.01.112 24629931

[14] Zhang H Xu Y Yu S. Co-production of functional xylooligosaccharides and fermentable sugars from corncob with effective acetic acid prehydrolysis. Bioresour Technol. (2017) 234:343–9. 10.1016/j.biortech.2017.02.094 28340439

[15] Zhang H Zhou X Xu Y Yu S. Production of xylooligosaccharides from waste xylan, obtained from viscose fiber processing, by selective hydrolysis using concentrated acetic acid. J Wood Chem Technol. (2017) 37:1–9. 10.1080/02773813.2016.1214154

[16] Samanta A Chikkerur J Roy S Kolte A Sridhar M Dhali A. Xylooligosaccharides production from tobacco stalk xylan using edible acid. Curr Sci. (2019) 117:1521–5. 10.18520/cs/v117/i9/1521-1525

[17] Ying W Xu Y Zhang J. Effect of sulfuric acid on production of xylooligosaccharides and monosaccharides from hydrogen peroxide-acetic acid-pretreated poplar. Bioresour Technol. (2021) 321:124472. 10.1016/j.biortech.2020.124472 33307483

[18] De Freitas C Carmona E Brienzo M. Xylooligosaccharides production process from lignocellulosic biomass and bioactive effects. Bioact Carbohydr Diet Fibre. (2019) 18:100184. 10.1016/j.bcdf.2019.100184

[19] Jnawali P Kumar V Tanwar B Hirdyani H Gupta P. Enzymatic production of xylooligosaccharides from brown coconut husk treated with sodium hydroxide. Waste Biomass Valori. (2017) 9:1757–66. 10.1007/s12649-017-9963-4

[20] Teleman A Lundqvist J Tjerneld F Stålbrand H Dahlman O. Characterization of acetylated 4-O-methylglucuronoxylan isolated from aspen employing 1H and 13C NMR spectroscopy. Carbohydr Res. (2000) 329:807–15. 10.1016/S0008-6215(00)00249-4 11125823

[21] Arai T Biely P Uhliarikova I Sato N Makishima S Mizuno M. Structural characterization of hemicellulose released from corn cob in continuous flow type hydrothermal reactor. J Biosci Bioeng. (2019) 127:222–30. 10.1016/j.jbiosc.2018.07.016 30143337

[22] Linares-Pasten JA Aronsson A Karlsson EN. Structural considerations on the use of endo-xylanases for the production of prebiotic xylooligosaccharides from biomass. Curr Protein Pept Sci. (2018) 19:48–67. 10.2174/1389203717666160923155209 27670134 PMC5738707

[23] Dilokpimol A Nakai H Gotfredsen CH Appeldoorn M Baumann MJ Nakai N. Enzymatic synthesis of beta-xylosyl-oligosaccharides by transxylosylation using two beta-xylosidases of glycoside hydrolase family 3 from Aspergillus nidulans FGSC A4. Carbohydr Res. (2011) 346:421–9. 10.1016/j.carres.2010.12.010 21215963

[24] Kurakake M Fujii T Yata M Okazaki T Komaki T. Characteristics of transxylosylation by β-xylosidase from aspergillus awamori K4. Biochim Biophys Acta. (2005) 1726:0–279. 10.1016/j.bbagen.2005.08.009 16202538

[25] Kim YW Fox DT Hekmat O Kantner T Mcintosh LP Warren R. Glycosynthase-based synthesis of xylo-oligosaccharides using an engineered retaining xylanase from cellulomonas fimi. Org Biomol Chem. (2006) 4:2025–20. 10.1039/b601667g 16688347

[26] Yang R Xu S Wang Z Yang W. Aqueous extraction of corncob xylan and production of xylooligosaccharides. LWT Food Sci Technol. (2005) 38:677–82. 10.1016/j.lwt.2004.07.023

[27] Liu X Liu Y Jiang Z Liu H Yang S Yan Q. Biochemical characterization of a novel xylanase from paenibacillus barengoltzii and its application in xylooligosaccharides production from corncobs. Food Chem. (2018) 264:310–8. 10.1016/j.foodchem.2018.05.023 29853381

[28] Seesuriyachan P Kawee-Ai A Chaiyaso T. Green and chemical-free process of enzymatic xylooligosaccharide production from corncob: enhancement of the yields using a strategy of lignocellulosic destructuration by ultra-high pressure pretreatment. Bioresour Technol. (2017) 241:537–44. 10.1016/j.biortech.2017.05.193 28601771

[29] Boonchuay P Techapun C Leksawasdi N Seesuriyachan P Hanmoungjai P Watanabe M. An integrated process for xylooligosaccharide and bioethanol production from corncob. Bioresour Technol. (2018) 256:399–407. 10.1016/j.biortech.2018.02.004 29475148

[30] Xue JL Zhao S Liang RM Yin X Jiang SX Su LH. A biotechnological process efficiently co-produces two high value-added products, glucose and xylooligosaccharides, from sugarcane bagasse. Bioresour Technol. (2016) 204:130–8. 10.1016/j.biortech.2015.12.082 26773956

[31] Reddy SS Krishnan C. Production of high-pure xylooligosaccharides from sugarcane bagasse using crude β-xylosidase-free xylanase of bacillus subtilis KCX006 and their bifidogenic function. LWT Food Sci Technol. (2016) 65:237–45. 10.1016/j.lwt.2015.08.013

[32] Zhou X Zhao J Zhang X Xu Y. An eco-friendly biorefinery strategy for xylooligosaccharides production from sugarcane bagasse using cellulosic derived gluconic acid as efficient catalyst. Bioresour Technol. (2019) 289:121755. 10.1016/j.biortech.2019.121755 31301946

[33] Zhou X Xu Y. Integrative process for sugarcane bagasse biorefinery to co-produce xylooligosaccharides and gluconic acid. Bioresour Technol. (2019) 282:81–7. 10.1016/j.biortech.2019.02.129 30852335

[34] Faryar R Linares-Pastén JA Immerzeel P Mamo G Andersson M Stålbrand H. Production of prebiotic xylooligosaccharides from alkaline extracted wheat straw using the K80R-variant of a thermostable alkali-tolerant xylanase. Food Bioprod Process. (2015) 93:1–10. 10.1016/j.fbp.2014.11.004

[35] Romero-Fernandez M Moreno-Perez S Martins De Oliveira S Santamaria RI Guisan JM Rocha-Martin J. Preparation of a robust immobilized biocatalyst of beta-1,4-endoxylanase by surface coating with polymers for production of xylooligosaccharides from different xylan sources. N Biotechnol. (2018) 44:50–8. 10.1016/j.nbt.2018.04.007 29704649

[36] Huang C Lai C Wu X Huang Y He J Huang C. An integrated process to produce bio-ethanol and xylooligosaccharides rich in xylobiose and xylotriose from high ash content waste wheat straw. Bioresour Technol. (2017) 241:228–35. 10.1016/j.biortech.2017.05.109 28570888

[37] Sophonputtanaphoca S Pridam C Chinnak J Nathong M Juntipwong P. Production of non-digestible oligosaccharides as value-added by-products from rice straw. Agric Nat Resour. (2018) 52:169–75. 10.1016/j.anres.2018.06.013

[38] Khat-Udomkiri N Sivamaruthi BS Sirilun S Lailerd N Peerajan S Chaiyasut C. Optimization of alkaline pretreatment and enzymatic hydrolysis for the extraction of xylooligosaccharide from rice husk. AMB Express. (2018) 8:115. 10.1186/s13568-018-0645-9 30014174 PMC6047951

[39] Banerjee S Patti AF Ranganathan V Arora A. Hemicellulose based biorefinery from pineapple peel waste: xylan extraction and its conversion into xylooligosaccharides. Food Bioprod Process. (2019) 117:38–50. 10.1016/j.fbp.2019.06.012

[40] Palaniappan A Balasubramaniam VG Antony U. Prebiotic potential of xylooligosaccharides derived from finger millet seed coat. Food Biotechnol. (2017) 31:264–80. 10.1080/08905436.2017.1369433

[41] Aachary AA. Prebiotics: specific colonic nutrients. In: Prapulla SG, editor. Bioactive Xylooligosaccharides From Corncob: Enzymatic Production and Applications (Thesis) submitted to Univ. Of Mysore. Mysore: Aacharya, A. A. (2009). p. 19.

[42] Durack DT Glauser MP. The inflammatory cytokines - new developments in the pathophysiology and treatment of septic shock - discussion. Drugs. (1996) 52:17. 10.2165/00003495-199600522-00004 8869831

[43] Childs CE Roytio H Alhoniemi E Fekete AA Forssten SD Hudjec N. Xylo-oligosaccharides alone or in synbiotic combination with bifidobacterium animalis subsp. Lactis induce bifidogenesis and modulate markers of immune function in healthy adults: a double-blind, placebo-controlled, randomised, factorial cross-over study. Br J Nutr. (2014) 111:1945–56. 10.1017/S0007114513004261 24661576

[44] Chen H Chen Y Chang H. Immunomodulatory effects of xylooligosaccharides[J]. Food Sci Technol Res. (2012) 18:195–9. 10.3136/fstr.18.195

[45] Hansen CH Frokiaer H Christensen AG Bergstrom A Licht TR Hansen AK. Dietary xylooligosaccharide downregulates IFN-gamma the low-grade inflammatory cytokine IL-1beta systemically in mice. J Nutr. (2013) 143:533–40. 10.3945/jn.112.172361 23427328

[46] Nabarlatz D Montane D Kardosova A Bekesova S Hribalova V Ebringerova A. Almond shell xylo-oligosaccharides exhibiting immunostimulatory activity. Carbohydr. Res. (2007) 342:1122–8. 10.1016/j.carres.2007.02.017 17362891

[47] Lecerf J.-M. Dépeint F Clerc E Dugenet Y Niamba CN. Xylo-oligosaccharide (XOS) in combination with inulin modulates both the intestinal environment and immune status in healthy subjects, while XOS alone only shows prebiotic properties. Br J Nutr. (2012) 108:1847–58. 10.1017/S0007114511007252 22264499

[48] Howe GR Benito E Castelleto R Cornée J Estève J Gallagher RP. Dietary intake of fiber decreased risk of cancers of the colon rectum: evidence from the combined analysis of 13 case-control studies. Jnci J Natl Cancer. (1992) 84:1887–96. 10.1093/jnci/84.24.1887 1334153

[49] Ando H Ohba H Sakaki T Takamine K Kamino Y Moriwaki S. Hot-compressed-water decomposed products from bamboo manifest a selective cytotoxicity against acute lymphoblastic leukemia cells. Toxicol In Vitro. (2004) 18:765–71. 10.1016/j.tiv.2004.03.011 15465641

[50] Maeda R Ida T Ihara H Sakamoto T. Induction of apoptosis in MCF-7 cells by beta-1,3-xylooligosaccharides prepared from caulerpa lentillifera. Biosci Biotech Bioch. (2012) 76:1032–4. 10.1271/bbb.120016 22738982

[51] Aacharya AA Gobinatha D Srinivasanb K Prapulla SG. Protective effect of xylooligosaccharides from corncob on 1,2-dimethylhydrazine induced colon cancer in rats[J]. Bioact Carbohydr Diet Fibre. (2015) 5:146–52. 10.1016/j.bcdf.2015.03.004

[52] Yu X Yin J Li L Luan C Li S. Prebiotic potential of xylooligosaccharides derived from corn cobs their *In Vitro* antioxidant activity when combined with lactobacillus. J Microbiol Biotechnol. (2015) 25:1084–92. 10.4014/jmb.1501.01022 25791856

[53] Rashad MM Mahmoud AE Nooman MU Mahmoud HA Keshta AT. Production of antioxidant xylooligosaccharides from lignocellulosic materials using bacillus amyloliquifaciens NRRL B-14393 xylanase. J App Pharm Sci. (2016) 6:30–6. 10.7324/JAPS.2016.60606

[54] Gowdhaman D Ponnusami V. Production and optimization of xylooligosaccharides from corncob by bacillus aerophilus KGJ2 xylanase and its antioxidant potential. Int J Biol Macromol. (2015) 79:595–600. 10.1016/j.ijbiomac.2015.05.046 26038103

[55] Huang D Ou B Prior RL. The chemistry behind antioxidant capacity assays. J Agric Food Chem. (2005) 53:1841–56. 10.1021/jf030723c 15769103

[56] Jagtap S Deshmukh RA Menon S Das S. Xylooligosaccharides production by crude microbial enzymes from agricultural waste without prior treatment and their potential application as nutraceuticals. Bioresour Technol. (2017) 245:283–8. 10.1016/j.biortech.2017.08.174 28892703

[57] Bouiche C Boucherba N Benallaoua S Martinez J Diaz P Pastor FIJ. Differential antioxidant activity of glucuronoxylooligosaccharides (UXOS) and arabinoxylooligosaccharides (AXOS) produced by two novel xylanases. Int J Biol Macromol. (2019) 155:1075–83. 10.1016/j.ijbiomac.2019.11.073 31712139

[58] Valls C Pastor FJ Vidal T Roncero MB Díaz P Martínez J. Antioxidant activity of xylooligosaccharides produced from glucuronoxylan by Xyn10A and Xyn30D xylanases and eucalyptus autohydrolysates. Carbohydr Polym. (2018) 194:43–50. 10.1016/j.carbpol.2018.04.028 29801857

[59] Hsu CK Liao JW Chung YC Hsieh CP Chan YC. Xylooligosaccharides fructooligosaccharides affect the intestinal microbiota precancerous colonic lesion development in rats. J Nutr. (2004) 134:1523–8. 10.1093/jn/134.6.1523 15173423

[60] Palframan RJ Gibson GR Rastall RA. Carbohydrate preferences of bifidobacterium species isolated from the human gut. Curr Issues Intest Microbiol. (2003) 4:71–5. PMID: 14503691 14503691

[61] Pan X Wu T Zhang L Cai L Song Z. Influence of oligosaccharides on the growth and tolerance capacity of lactobacilli to simulated stress environment. Lett Appl Microbiol. (2009) 48:362–7. 10.1111/j.1472-765X.2008.02539.x 19187509

[62] Ebersbach T Andersen JB Bergstr MA Hutkins RW Licht TR. Xylo-oligosaccharides inhibit pathogen adhesion to enterocytes *in vitro*. Res Microbiol. (2012) 163:22–7. 10.1016/j.resmic.2011.10.003 22056968

[63] Dotsenko G Meyer AS Canibe N Thygesen A Nielsen MK Lange L. Enzymatic production of wheat and ryegrass derived xylooligosaccharides and evaluation of their in vitro effect on pig gut microbiota. Biomass Convers Bior. (2017) 8:497–507. 10.1007/s13399-017-0298-y

[64] Chen Y Xie Y Zhong R Liu L Lin C Xiao L. Effects of xylo-oligosaccharides on growth and gut microbiota as potential replacements for antibiotic in weaning piglets. Front Microbiol. (2021) 12:641172. 10.3389/fmicb.2021.641172 33717037 PMC7947891

[65] Yin J Li F Kong X Wen C Guo Q Zhang L. Dietary xylo-oligosaccharide improves intestinal functions in weaned piglets. Food Funct. (2019) 10:2701–9. 10.1039/C8FO02485E 31025998

[66] Yuan L Li W Huo Q Du C Wang Z Yi B. Effects of xylo-oligosaccharide and flavomycin on the immune function of broiler chickens. PeerJ. (2018) 6:e4435. 10.7717/peerj.4435 29527412 PMC5842763

[67] Suo HQ Lin L Xu GH Xiao L Chen XG Xia RR. Effectiveness of dietary xylo-oligosaccharides for broilers fed a conventional corn-soybean meal diet. J Integr Agr. (2015) 14:2050–7. 10.1016/S2095-3119(15)61101-7

[68] De Maesschalck C Eeckhaut V Maertens L De Lange L Marchal L Nezer C. Effects of xylo-oligosaccharides on broiler chicken performance and microbiota. Appl Environ Microbiol. (2015) 81:5880–8. 10.1128/AEM.01616-15 26092452 PMC4551243

[69] Li DD Ding XM Zhang KY Bai SP Wang JP Zeng QF. Effects of dietary xylooligosaccharides on the performance, egg quality, nutrient digestibility and plasma parameters of laying hens. Anim Feed Sci Tech. (2017) 225:20–6. 10.1016/j.anifeedsci.2016.12.010

[70] Schokker D Jansman AJ Veninga G De Bruin N Vastenhouw SA De Bree FM. Perturbation of microbiota in one-day old broiler chickens with antibiotic for 24 hours negatively affects intestinal immune development. BMC Genomics. (2017) 18:241. 10.1186/s12864-017-3625-6 28320307 PMC5359956

[71] Chen Y Xie Y Zhong R Han H Liu L Chen L. Effects of graded levels of xylo-oligosaccharides on growth performance, serum parameters, intestinal morphology and intestinal barrier function in weaned piglets. J Anim Sci. (2021) 99:skab183. 10.1093/jas/skab183 34097723 PMC8280928

[72] Zhu LH Zhao KL Chen XL Xu JX. Impact of weaning and an antioxidant blend on intestinal barrier function and antioxidant status in pigs. J Anim Sci. (2012) 90:2581–9. 10.2527/jas.2011-4444 22896732

[73] Wang J Cao Y Wang C Sun B. Wheat bran xylooligosaccharides improve blood lipid metabolism and antioxidant status in rats fed a high-fat diet. Carbohydr Polym. (2011) 86:1192–7. 10.1016/j.carbpol.2011.06.014

[74] Guerreiro I Couto A Pérez-Jiménez A Oliva-Teles A Enes P. Gut morphology and hepatic oxidative status of European sea bass (Dicentrarchus labrax) juveniles fed plant feedstuffs or fishmeal-based diets supplemented with short-chain fructo-oligosaccharides and xylo-oligosaccharides. Br J Nutr. (2015) 114:1975–84. 10.1017/S0007114515003773 26435350

[75] Ebersbach T Jorgensen JB Heegaard PM Lahtinen SJ Ouwehand AC Poulsen M. Certain dietary carbohydrates promote Listeria infection in a guinea pig model, while others prevent it. Int J Food Microbiol. (2010) 140:218–24. 10.1016/j.ijfoodmicro.2010.03.030 20417983

[76] Pourabedin M Guan L Zhao X. Xylo-oligosaccharides and virginiamycin differentially modulate gut microbial composition in chickens. Microbiome. (2015) 3:15. 10.1186/s40168-015-0079-4 25874109 PMC4396176

[77] Kravtsov E Yermolayev A Anokhina I Yashina N Chesnokova V Dalin M. Adhesion characteristics of lactobacillus is a criterion of the probiotic choice. B Exp Biol Med. (2008) 145:232–4. 10.1007/s10517-008-0058-x 19023977

[78] Belenguer A Duncan SH Holtrop G Anderson SE Lobley GE Flint HJ. Impact of pH on lactate formation and utilization by human fecal microbial communities. Appl Environ Microbiol. (2007) 73:6526–33. 10.1128/AEM.00508-07 17766450 PMC2075063

